# Technical feasibility of novel immunostimulatory low‐dose radiation for polymetastatic disease with CBCT‐based online adaptive and conventional approaches

**DOI:** 10.1002/acm2.14303

**Published:** 2024-02-20

**Authors:** Nour Nasser, Bradford A. Perez, Jose A. Penagaricano, Jimmy J. Caudell, Daniel E. Oliver, Kujtim Latifi, Eduardo G. Moros, Gage Redler

**Affiliations:** ^1^ Department of Radiation Oncology Moffitt Cancer Center Tampa Florida USA; ^2^ Department of Physics University of South Florida Tampa Florida USA

**Keywords:** adaptive therapy, development (new technology and techniques), treatment planning/virtual clinical studies

## Abstract

**Purpose:**

A workflow/planning strategy delivering low‐dose radiation therapy (LDRT) (1 Gy) to all polymetastatic diseases using conventional planning/delivery (Raystation/Halcyon = “conventional”) and the AI‐based Ethos online adaptive RT (oART) platform is developed/evaluated.

**Methods:**

Using retrospective data for ten polymetastatic non‐small cell lung cancer patients (5‐52 lesions each) with PET/CTs, gross tumor volumes (GTVs) were delineated using PET standardized‐uptake‐value (SUV) thresholding. A 1 cm uniform expansion of GTVs to account for setup/contour uncertainty and organ motion‐generated planning target volumes (PTVs). Dose optimization/calculation used the diagnostic CT from PET/CT. Dosimetric objectives were: D_min,0.03cc_ ≥ 95% (acceptable variation (Δ) ≥ 90%), V_100%_ ≥ 95% (Δ ≥ 90%), and D_0.03cc_ ≤ 120% (Δ ≤ 125%). Additionally, online adaptation was simulated. When available, subsequent diagnostic CT was used to represent on‐treatment CBCT. Otherwise, the CT from PET/CT used for initial planning was deformed to simulate clinically representative changes.

**Results:**

All initial plans generated, both for Raystation and Ethos, achieved clinical goals within acceptable variation. For all patients, D_min,0.03cc_ ≥ 95%, V_100%_ ≥ 95%, and D_0.03cc_ ≤ 120% goals were achieved for 84.8%/99.5%, 97.7%/98.7%, 97.4%/92.3%, in conventional/Ethos plans, respectively. The ratio of 50% isodose volume to PTV volume (R_50%_), maximum dose at 2 cm from PTV (D_2cm_), and the ratio of the 100% isodose volume to PTV volume (conformity index) in Raystation/Ethos plans were 7.9/5.9; 102.3%/88.44%; and 0.99/1.01, respectively. In Ethos, online adapted plans maintained PTV coverage whereas scheduled plans often resulted in geographic misses due to changes in tumor size, patient position, and body habitus. The average total duration of the oART workflow was 26:15 (min:sec) ranging from 6:43 to 57:30. The duration of each oART workflow step as a function of a number of targets showed a low correlation coefficient for influencer generation and editing (*R* ^2 ^= 0.04 and 0.02, respectively) and high correlation coefficient for target generation, target editing and plan generation (*R* ^2 ^= 0.68, 0.63 and 0.69, respectively).

**Conclusions:**

This study demonstrates feasibility of conventional planning/treatment with Raystation/Halcyon and highlights efficiency gains when utilizing semi‐automated planning/online‐adaptive treatment with Ethos for immunostimulatory LDRT conformally delivered to all sites of polymetastatic disease.

## 1 INTRODUCTION

Treatment of metastatic non‐small cell lung cancer (NSCLC) has historically relied on chemotherapy, with a low long‐term survival rate.^1^ Recent studies show that immunotherapy alone^2^, ^3^ or in combination with chemotherapy^4^, ^5^, ^6^ improved overall survival for metastatic NSCLC patients. Combination of ipilimumab and nivolumab for patients with metastatic or recurrent PD‐L1 positive NSCLC showed improvement in overall response rate, response duration, and overall survival compared to those who received chemotherapy.^7^ Thus, the FDA approved ipilimumab and nivolumab as first‐line therapy for NSCLC patients with PD‐L1 expression ≥1%. However, only 35.9% of patients with PD‐L1≥1% receiving ipilimumab and nivolumab showed an objective response.^7^ Studies showed that RT is able to improve outcomes for metastatic NSCLC patients,^8^ improve progression‐free survival and overall survival,^9^ and enhance immunotherapy efficacy.^10^ Evidence indicates that low‐dose radiotherapy (LDRT) can affect macrophage differentiation and improve the migration of T cells to the tumor microenvironment,^11^ which will improve immunotherapy efficacy by stimulating anti‐tumor immune response.^12^ Furthermore, LDRT reprograms the microenvironment of tumors with low to no immune infiltration by reversing tumor immune desertification and resistance to immunotherapy.^13^

Treating all sites of polymetastatic disease periodically with a conventional treatment planning system (TPS) as well as a novel CBCT‐guided online adaptative radiotherapy (oART) system was investigated. The conventional planning was based on the Raystation TPS with Halcyon delivery, using a clinically commissioned model.^14^ While the Ethos oART system provided an alternative with semi‐automated planning and a streamlined online adaptation that will account for changes in patient anatomy aligned with immunotherapy administration efficiently. The newly developed Ethos (Varian Medical Systems, Inc., Palo Alto, CA) cone beam CT (CBCT)‐based ring‐gantry oART system is based on the Varian Halcyon treatment machine with a 6 MV flattening filter free (FFF) single beam energy, a faster gantry rotation (up to 4 RPM), and dual‐stacked and staggered multi‐leaf collimators without collimating jaws. Ethos also provides rapid iterative reconstruction for high‐quality kV‐CBCT,^15^ with contrast and noise similar to that of a CT simulator. The novel planning paradigm introduced by Ethos efficiently auto‐generates initial treatment plans and provides an efficient online adaptive platform to account for changes during the treatment course, a particularly useful feature to account for patient changes in the context of delivering low‐dose radiotherapy (LDRT) on a prolonged time‐scale similar to that of systemic immunotherapy administration (i.e., weeks to months between fractions). Using physician clinical goals, the Ethos intelligent optimization engine (IOE) automatically generates the optimization objective function for the photon optimization (PO) algorithm.^16^ To ensure high quality dose distribution to targets and control monitor units (MU) and normal tissue goals, the IOE generates helper structures and helper objectives.^16^ Clinical goals could be assigned four priority levels depending on the importance from P1 (most important) to P4 (least important) as well as an “R” priority to simply report a dosimetric value for evaluation. The IOE creates new non‐overlapping structures and reassigns weights for the clinical goals to help achieve high‐quality dose distributions based on goal priority levels.

Online adaptation in Ethos uses the synthetic CT, deformable image registration (DIR) of the initial planning CT to the CBCT scan of the day, for dose calculation.^16^, ^17^ Influencer structures, a subset of treatment site‐specific OARs, are used for structure‐guided DIR to generate targets and remaining OARs. Additionally, AI‐based auto‐segmentation is available for initial planning for pelvic and abdomen regions (Ethos v1.1). During the adaptive process, auto‐segmented targets and OARs with high priority (P1 and P2) are reviewed and edited, while remaining structures are only visible after treatment delivery. Ethos generates a scheduled plan, initial plan calculated on the anatomy of the day, and an adapted plan that has the same beam geometry and clinical goals as the initial plan but is re‐optimized to adapt the dose distribution based on the patient's anatomy of the day.^17^

The aim of this study was to develop an efficient workflow and evaluate the feasibility of treating all sites of gross disease with LDRT (1 Gy) for polymetastatic NSCLC patients to boost immunotherapy response using the conventional planning and the newly developed Ethos oART, allowing for plan adaptation to account for anatomy change during immunotherapy administration. This study developed an algorithmic approach to conformally and comprehensively treat widespread polymetastatic disease (up to 52 separate lesions investigated in this work) with LDRT using conventional planning and AI‐based Ethos oART. Online adaptive sessions were simulated in Ethos to evaluate the capability and utility of adapting such plans to account for changes in patient anatomy and evolving metastatic disease during an extended treatment course aligned with periodic systemic immunotherapy administration. Evaluation of feasibility and dosimetric quality of initial and online adaptive planning for polymetastatic lung cancer with the Ethos system was performed. Besides the conventional plans representing what could potentially be used for non‐adaptive treatment of polymetastatic disease, they also served as a proof of overall dosimetric feasibility of conformally treating widespread disease with LDRT as well as a dosimetric comparison to evaluate Ethos initial plan quality and determine an optimal Ethos planning approach. Planning within the Ethos paradigm using clinical goals is crucial for online adaptation of Ethos initial plans for subsequent treatment fractions to efficiently account for disease response/progression and patient anatomical changes.

## 2 MATERIALS AND METHODS

Retrospective data for ten patients with polymetastatic NSCLC were used in this study. FDG PET/CT scans were used for delineation of gross tumor volumes (GTVs) using PET standardized‐uptake‐value (SUV). Quantitative evaluation and contouring of PET scans were performed in Mirada (Mirada Medical Ltd., Oxford, UK), where normal liver was contoured with the SUV mean+2𝜎 (𝜎: standard deviation) considered as a lower threshold to initially define all GTVs. Targets were separated, reviewed, and edited by the radiation oncologist. Planning target volumes (PTVs) were generated with a 1 cm expansion of GTVs to account for contouring and setup uncertainty as well as organ motion. Number of targets per patient varied from 5 to 52 (median of 15) for a total of 196 across all ten patients. For initial plan quality comparison, two initial plans were created for each patient: one in a conventional TPS that utilizes standard optimization objectives to tailor a cost function using Monte Carlo dose calculation (Raystation v9B, RaySearch Laboratories AB, Stockholm, Sweden) and one in the novel Ethos platform that utilizes clinical goals and semi‐automated plan generation using Acuros XB linear Boltzmann transport equation solver dose calculation. The concept of low dose irradiation is not new, but clinical efficacy of different low dose prescriptions is still somewhat unknown. In this work, prescribed target dose is 1 Gy, based on what has been used in related work in the literature.^13^, ^18^ For this work, an Ethos emulator software package (v1.1) was utilized. This provided the Ethos TPS with initial planning and capabilities to simulate the online adaptive workflow in silico without requiring a fully functioning clinical system. Using this Ethos emulator, in addition to initial plans, online adapted plans were generated and evaluated. Dosimetric advantages of online adaptation were assessed via comparison to “scheduled” plan dosimetry (i.e., recalculation of initial plan on updated patient anatomy of the day that includes variable response/progression across polymetastatic lesions, representing the option to deliver the non‐adapted Ethos plan at treatment). The timing efficiency of the developed Ethos workflow for such treatments is also analyzed.

### 2.1 Conventional planning

The CT datasets associated with the PET/CT and the GTVs delineated in Mirada were imported into Raystation (v9B) for plan generation using a conventional optimization approach and Monte Carlo dose calculation. PTVs were created with 1 cm expansion from the GTVs.

**FIGURE 1:**
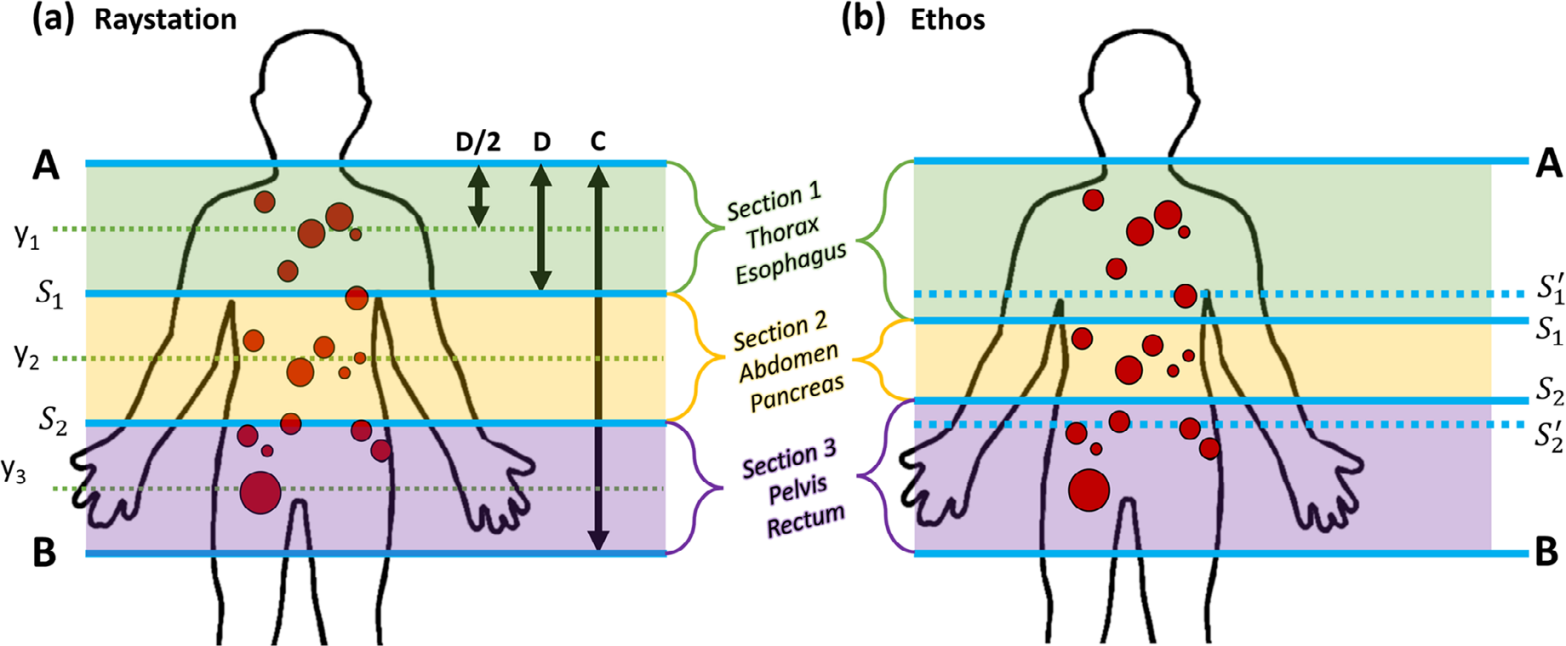
(a) Definition of number and location of isocenters, and treatment sections for each patient with standard planning/treatment approaches (Halcyon/Raystation). A/B are the most superior/inferior slices with PTV+1cm. D is the length of each section. S_x_ is the limit between two adjacent sections. Y_x_ is the location (superior/inferior) of isocenters. (b) Definition of body sections and associated anatomical region (RT intent) in Ethos based on target locations and maximum length of combined targets in a single RT intent. Dashed lines (S′_1_, S′_2_) are initial body section division based on the location of targets as detailed in (a). The solid lines (S_1_, S_2_) are the final body section division adjusting for specific target locations near section junctions.

A systematic approach was used for defining isocenter locations based on the number and distribution of targets. The patient region containing targets was first sectioned evenly in the superior/inferior direction, guided by Halcyon/Ethos maximum field size limitations of 28 cm (Figure 1a). The length of the total treatment area (C) was defined based on the most superior/inferior PTV with 1 cm margin added in both the superior and inferior directions (to ensure sufficient margin at field border to provide target coverage). These superior and inferior borders are shown in Figure 1a as “A” and “B,” respectively. Therefore, C = A—B. The number of isocenters was defined as N = C/22 cm (with 22 cm used as a conservative incorporation of the true 28 cm limitation). All sections were the same length (D), given by the total treatment area divided by the number of isocenters (D = C/N). The inferior limit (S_n_) of each section was defined as the superior limit plus the length of the section (S_1 _= A+D, S_2 _= S_1_+D, etc.). All isocenters were located at the center of each section in the superior/inferior direction, with this location (Y_n_) given by the superior limit of the section plus half of the section length (Y_1 _= A+D/2, Y_2 _= S_1_+D/2, etc.). The location of all isocenters in the lateral and anterior/posterior directions was defined by the centroid for all PTVs to avoid complex shifts during treatment delivery (i.e., limiting shifts between isocenters to table in/out movements only).

The standard planning approach was used based on optimization objectives to achieve clinical goals for all PTVs while sparing normal tissue (Table 1). One or two arcs (Halcyon, 6MV FFF) were created for each isocenter with different collimator angles to help the optimizer achieve conformal target coverage. The following planning structures were created: combination of all PTVs (All_PTVs), external structure excluding All_PTVs (Normal tissue) and external structure excluding All_PTVs expanded by 2 cm (Normal tissue_2 cm). The initial plan optimization was based on the optimization function of the above structures in addition to the external structure to achieve conformal coverage. When optimizing on All_PTVs resulted in failed goals, additional PTV‐specific optimization objectives were used to tailor the final plan coverage/homogeneity for these specific PTVs.

**TABLE 1:** PTV clinical goals and acceptable variations.

PTV clinical goals	Acceptable variation
V_1Gy_ ≥ 95%	V_1Gy_ ≥ 90%
D_min,0.03cc_ ≥ 95%	D_min,0.03cc_ ≥ 90%
D_0.03cc_ ≤ 120%	D_0.03cc_ ≤ 125%

*Note*: V_1Gy_, D_min,0.03cc_, and D_0.03cc_ are the PTV volume receiving 1 Gy, minimum dose to all but 0.03cc, and maximum dose to a 0.03cc voxel within the PTV, respectively. Dose percentages are with respect to prescription dose of 1 Gy.

### 2.2 Ethos planning

#### 2.2.1 Initial planning

Similar to planning in Raystation (see Conventional Planning section above), CT datasets of the PET/CT and associated structures delineated in Mirada were imported into Ethos RT intents (an Ethos‐specific concept combining elements of RT prescription, images/structures, planning goals, and resultant treatment plan) in lieu of traditional radiotherapy simulation CT for initial plan generation. PTVs were created in Ethos with the same 1 cm margin from GTVs via automated contour derivation rules. Based on the distribution of targets, PTVs were grouped in different anatomical regions (Figure 1b) that were selected based on the highest number of influencers associated during the online adaptive workflow (Table 2) to facilitate influencer structure‐guided DIR of potentially widely distributed metastatic targets within a particular body region. The maximum target length in a single RT intent for a single/dual isocenter was 26 cm/38.5 cm, respectively. In Figure 1b, dotted lines represented the initial RT intent boundaries (based on the algorithm outlined in Figure 1a) and solid lines represented the adjusted RT intent boundaries depending on PTV positions (to avoid scenarios with targets in more than one RT intent, as co‐optimization beyond two isocenters is currently not available in Ethos). RT intent templates for each anatomical site were used for plan generation. These templates contained prescription and clinical goals, for targets and normal tissue, based on user‐predefined rules that will allow structure propagation and automatic update during adaptation. The highest two priorities (P1‐P2) we used for PTV goals, and the lowest two priorities (P3‐P4) were used for normal tissue sparing (this is in combination with the built in automatic normal tissue optimization used by Ethos to enforce target conformality).

**TABLE 2:** Anatomical region/site and their associated influencers used for RT intent creation.

Anatomical region/site	Associated influencers
Thorax/Esophagus	Esophagus, heart, lung left, lung right and trachea
Abdomen/Pancreas	Duodenum, liver, pancreas, stomach
Pelvis/Rectum	Bladder and rectum
Extremities: arm and leg (when needed)	No influencers

In dose preview, preliminary dose calculation of the expected plans was generated based on a 9‐field IMRT plan calculated with Fourier transform dose calculation algorithm (rather than final plan beam geometry and calculation based on Acuros XB algorithm) with the display of isodose line distribution, dose‐volume‐histogram (DVH) and achieved clinical goals. In dose preview, fine tuning is available by re‐arranging relative clinical goal priorities with a fast plan optimization and real‐time dose calculation showing the relative effect of these changes. Dose preview is also a workspace to evaluate the need of additional goals that can be updated in the planning directives workspace. Plan authorization will allow the generation of up to 5 plans for non‐lateralized targets: 7/9/12 equally spaced fixed‐field IMRT and 2/3 full‐arc VMAT. For laterally distributed targets, two additional plans can be generated: one with 7 lateral IMRT fixed‐fields and one with 2 lateral partial VMAT arcs. Normalization to the combination of all PTVs D_min,0.03cc _= 95% was used for 50% of the RT intents to ensure this specific goal was achieved.

**FIGURE 2:**
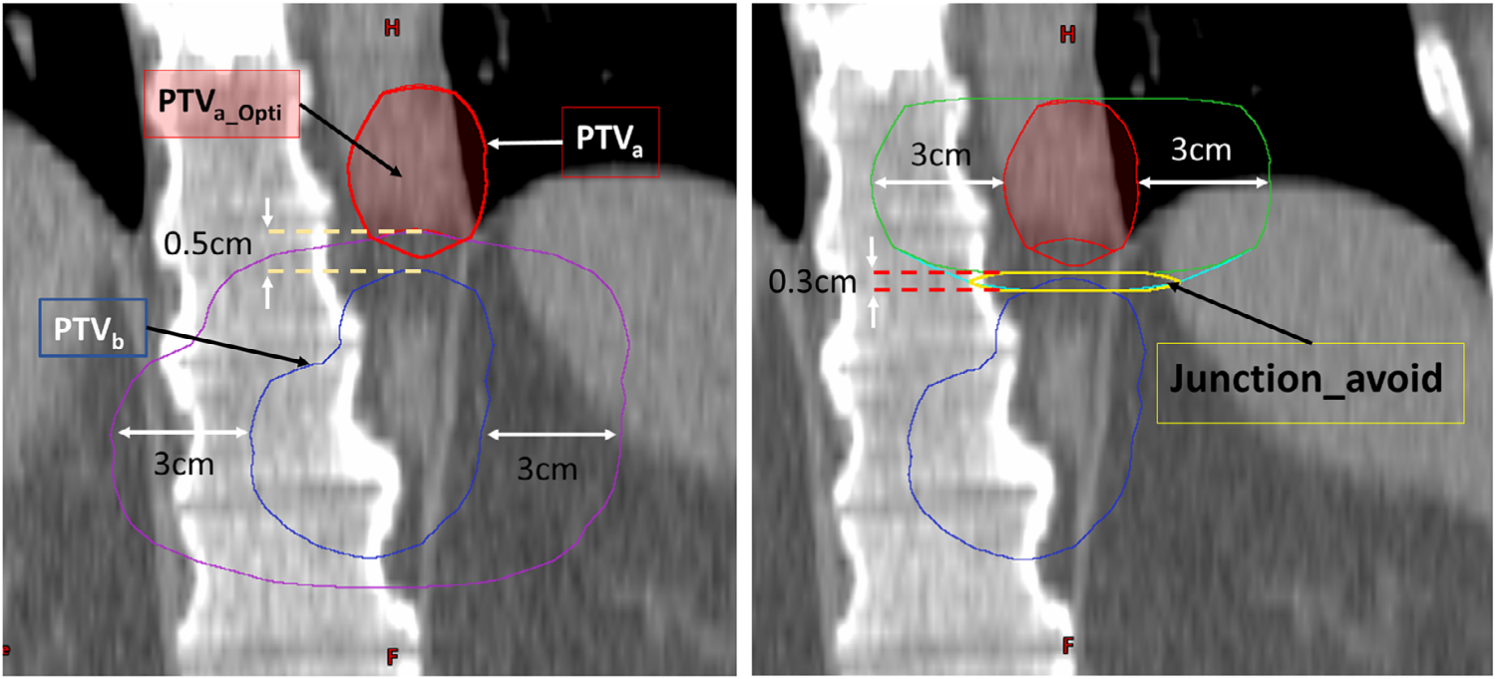
Structures created in the upper RT intent for the patient with junction problem that resulted in the best initial composite plan. PTV_a_ (red) and PTV_b_ (blue) are two adjacent targets in different RT intents. The goals used in the optimization are for PTV_a_Opti_ (shaded red), PTV_b_ and junction_avoid (yellow). Green/cyan and purple structures represent the expansion of the PTV_a_ and PTV_b_ to create the derived structures (junction_avoid and PTV_a_Opti_) used in the optimization, respectively.

One patient had a distribution of targets that did not allow for appreciable separation between targets in separate RT intents. This resulted in a junction problem, where two adjacent targets were in two different RT intents that could not be co‐optimized and therefore did not have knowledge of how the dose distribution from each would contribute to the other. An additional planning strategy to account for this particular patient was developed. Figure 2 shows structures created in the superior RT intent that resulted in the best composite plans when creating the same structures in the inferior RT intent. The goal of this planning approach was to create appropriate dosimetric gradients for targets in abutting patient sections with independently optimized beams so that the composite dose matched at this junction without appreciable hot or cold spots. This had to be done with anatomically‐derived helper structures so that these would update accordingly with changed anatomy during online plan adaptation. The planning approach was based on cropping the PTV_a_ (red) 0.5 cm away from the adjacent PTV_b_ (blue). This was done by expanding PTV_b_ 3 cm anteriorly/posteriorly/laterally and 0.5 cm towards the head/feet (purple). PTV_a_ is then cropped from this expansion of PTV_b_. The resulted cropped PTV_a_Opti_ (shaded red) was used in the optimization with the same PTV goals in Table 1 (V_100%_ ≥ 95% (P1), D_min,0.03cc_ ≥ 95% (P1), D_0.03cc_ ≤ 1 Gy (P2)). The adjacent target PTV_b_ was used as an avoidance structure with goals limiting the dose (D_0.03cc _< 0.5 Gy (P4) and V_1Gy_ < 2% (P4)). To limit hotspot between the two adjacent targets, an avoidance junction structure (“Junction_avoid”) was created (yellow) by cropping the expanded PTV_a_ (cyan, 3 cm laterally/anteriorly/posteriorly and 0.3 cm towards the feet) from the same previous expansion of PTV_a_ without the 0.3 cm expansion towards the feet (green) with the following goal applied: V_2Gy_ ≤ 10% (P2). This resulted in PTV_a_Opti_ and Junction_avoid structures that will be automatically updated when the disease is recontoured during oART.

Ethos v1.1 did not have tools to sum multiple plans or compare multiple plans simultaneously, therefore, all RT intents were exported to Eclipse (Varian Medical Systems, Palo Alto, CA) for composite dose generation and evaluation. In addition to clinical goals, the R_50%_, conformity index (CI) and D_2cm_ plan quality metrics were assessed. These were defined as follows: R_50%_ = $\frac{V_{50\%}}{V_{PTV}}$ and CI = $\frac{V_{100\%}}{V_{PTV}}$ and D_2cm_ is the maximum dose at 2 cm away from PTV (as % of prescribed 1 Gy), where *V*_50%_ is the volume getting 50% of the prescription dose, *V_PTV_* is the volume of All PTVs and *V*_100%_ is the volume getting 100% of the prescription dose.

#### 2.2.2 Online adaptive planning

Online adaptation was simulated for all RT intents for each patient. As these patients at this stage of their disease are generally not candidates for RT, radiation oncology simulation CTs and/or on‐treatment CBCTs were not available. Therefore, when simulating online adaptive treatment, which included anatomical variation representative of what would be seen when delivering the low‐dose RT every 2−6 weeks as proposed in this work, a substitute was used to represent on‐treatment CBCT. This substitute was either a subsequent diagnostic CT scan for the same patient (when available) or a low‐resolution deformation (to avoid unrealistic anatomical changes) of the CT (from the PET/CT) with a CT of different patient with similar positioning and body habitus. Influencer structures (Table 2), used for structure‐guided DIR and deformable propagation of targets and OARs from the initial scan to the scan of the day, were auto‐generated and manually edited by the radiation oncologist (when necessary). Underived targets such as GTVs were auto‐generated via structure‐guided DIR, reviewed, and edited by the radiation oncologist. PTVs and derived structures were adaptively auto updated based on the derivation rules used in the planning directives.

The system generated a scheduled plan, which is the initial plan re‐calculated on the anatomy of the day, and an adapted plan which utilizes beam geometry from the initial plan with re‐optimization based on initial clinical goals applied to structures of the day. The reference (initial plan on initial anatomy), scheduled, and adapted plans were calculated and their dosimetric metrics, isodose lines and DVH were available for evaluation and final selection of the superior plan. Offline dosimetric assessment of composite Ethos scheduled and adapted plans for all different RT intents for a given patient was performed in Eclipse, based on achieved clinical goals and normal tissue sparing characterized by R_50%_, D_2cm_ and CI. For the patient with adjacent targets in separate RT intents, the limitations of the approach used during online adaptation are investigated by evaluating composite dose distribution generated by the independent planning intents following adaptation and the capability to achieve clinical goals.

Timing data for the five different components, from influencer to plan generation, of the adaptive workflow were collected. To facilitate approximation of the time needed to complete the online adaptive workflow for a specific patient with a given number of targets, the duration for all oART steps was modelled as a function of the number of targets.

All data gathering and statistical tests were performed in Microsoft Excel (Microsoft, Redmond, WA). To statistically compare dosimetric indices for Ethos and Raystation plans a two‐sided student paired t‐test at 5% level was performed.

## 3 RESULTS

### 3.1 Initial plan quality

For traditional planning, the systematic approach used to determine the number and placement of isocenters was successfully applied to all patients. In the novel Ethos planning paradigm, the developed approach to separate targets for treatment in different RT intents was found straightforward and effective for 9 out of 10 patients. For one patient, complications were found related to the dose at the junction between two adjacent targets (separated by <5 mm in the superior‐inferior direction) in different independent RT intents. The approach developed to handle this scenario and generate appropriate dose gradients within each separate RT intent (see Figure 2) succeeded in achieving an initial composite plan that met all clinical goals and avoided junction hot spots. This was not an issue for traditional planning within Raystation as all isocenters could be simultaneously co‐optimized. Additionally, the lack of composite dose tools within Ethos made trial and error for designing appropriate junction structures/goals cumbersome as it required export of initial plans to a third‐party system for dose summation and evaluation with each variation to the approach.

**FIGURE 3:**
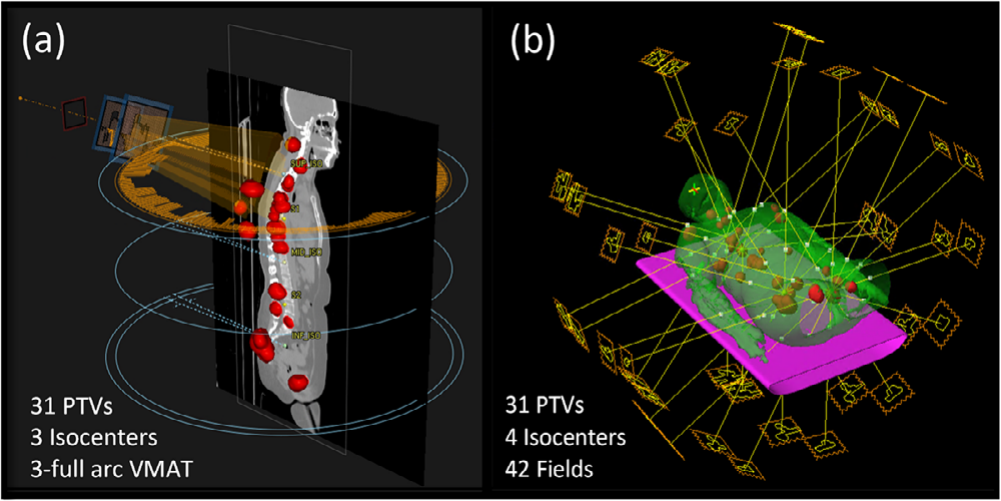
Initial plan set‐up for a patient with 31 PTVs (red) in (a) a conventional planning system (Raystation) and (b) in Ethos for OART.

In Ethos, fixed‐field IMRT plans were used in all RT intents due to higher plan quality compared to plans generated in conventional planning, where full‐arc VMAT plans were used. VMAT plans generated within Ethos were often found to violate clinical goals. Figure 3 shows the initial plan set‐up for a patient with 31 PTVs in (a) Raystation and (b) Ethos. The conventional plan used VMAT with 3 full‐arcs (Figure 3a). The Ethos plan (Figure 3b) consisted of three separate RT intents: thorax with 2 isocenters and 9 fields each for a total of 18 fixed‐field IMRT, abdomen with 12 fixed‐field IMRT, and pelvis with 12 fixed‐field IMRT for an overall total of 42 fixed‐field IMRT and 4 isocenters. Table 3 shows the percentage of PTVs and GTVs achieving clinical goals and the plan quality metrics for Ethos/Raystation initial plans. The percentage of PTVs/GTVs achieving each clinical goal are recorded for each individual plan and the values in Table 3 are an average of these percentages ± one standard deviation from the distribution of these percentages across all plans of a given type (Ethos initial, Raystation initial, Ethos scheduled, and Ethos adapted). For initial plans, both methods were similarly successful, with Ethos plans having a higher percentage of PTVs achieving clinical goals with V_1Gy_ ≥ 95%, D_min,0.03cc_ ≥ 0.95 Gy and D_max,0.03cc_ ≤ 1.20 Gy in Ethos/Raystation plans: 98.7 ± 4.2% / 95.7 ± 4.3%, 97.5 ± 6.3% / 76.8 ± 25.9%, and 86.2 ± 17.6% / 97.4 ± 5.6%, respectively. The difference was statistically significant and favorable for Ethos plans for D_min,0.03cc_ ≥ 0.95 Gy (*p* = 0.03). Goals for all PTVs in both Ethos and Raystation plans were achieved within the acceptable variations (i.e., V_1Gy_ ≥ 90%, D_min,0.03cc_ ≥ 0.90 Gy and D_max,0.03cc_ ≤ 1.25 Gy). As for the GTVs, all clinical goals were achieved for both Ethos and Raystation initial plans except for D_0.03cc_ ≤ 1.20 Gy with lower hotspot in Raystation plans represented by the percentage of GTVs achieving D_0.03cc_ ≤ 1.20 Gy in Ethos versus Raystation plans: 90 ± 18.6% versus 99.6 ± 1.2%. But D_0.03cc_ ≤ 1.25 Gy was achieved for all GTVs in both planning approaches for all patients. R_50%_ and D_2cm_ were lower in Ethos plans with 6.1 ± 1.9, and 88.4 ± 16.2%, respectively. CI was closer to the ideal value of 1.0 in Ethos plans versus Raystation plans (1.2 ± 0.1 vs. 1.4 ± 0.2). Therefore, Ethos plans were of higher quality and more conformal.

**TABLE 3:** The average percentage of PTVs and GTVs achieving clinical goals per plan ± one standard deviation and average achieved values for evaluation metrics (R_50%_, D_2cm_, CI) for Ethos/conventional TPS initial plans and for Ethos scheduled/adapted plans.

	Ethos initial plan	Raystation initial plan	Ethos scheduled plan	Ethos adapted plan
PTV V_1Gy_ ≥ 95%	98.7 ± 4.2%	95.7 ± 4.3%	28.5 ± 19.4%	91.8 ± 10.6%
PTV V_1Gy_ ≥ 90%	99.0 ± 4.2%	100.0 ± 1.56%	40.3 ± 27.7%	99.3 ± 2.1%
PTV D_min,0.03cc_ ≥ 0.95 Gy	97.5 ± 6.3%	76.8 ± 25.9%	10.2 ± 10.6%	86.0 ± 24.4%
PTV D_min,0.03cc_ ≥ 0.90 Gy	100.0 ± 0.0%	100.0 ± 1.2%	24.9 ± 16.9%	99.3 ± 2.1%
PTV D_0.03cc_ ≤ 1.20 Gy	86.2 ± 17.6%	97.4 ± 5.6%	80.9 ± 24.7%	91.6 ± 8.8%
PTV D_0.03cc_ ≤ 1.25 Gy	100.0 ± 0.0%	100.0 ± 0.0%	92.6 ± 15.9%	98.7 ± 4.2%
GTV V_1Gy_ ≥ 95%	100.0 ± 0.0%	100.0 ± 0.0%	67.8 ± 35.1%	94.8 ± 14.2%
GTV V_1Gy_ ≥ 90%	100.0 ± 0.0%	100.0 ± 0.0%	70.6 ± 35.8%	100.0 ± 0.0%
GTV D_min,0.03cc_ ≥ 0.95 Gy	100.0 ± 0.0%	100.0 ± 0.0%	74.6 ± 32.7%	100.0 ± 0.0%
GTV D_min,0.03cc_ ≥ 0.90 Gy	100.0 ± 0.0%	100.0 ± 0.0%	77.8 ± 33.2%	100.0 ± 0.0%
GTV D_0.03cc_ ≤ 1.20 Gy	90.0 ± 18.6%	99.6 ± 1.2%	82.7 ± 27.2%	93.9 ± 8.3%
GTV D_0.03cc_ ≤ 1.25 Gy	100.0 ± 0.0%	100.0 ± 0.0%	94.0 ± 19.0%	100.0 ± 0.0%
R_50%_	6.1 ± 1.9	7.9 ± 2.6	6.6 ± 2.1	5.9 ± 2.0
D_2cm_	88.4 ± 16.2%	102.7 ± 7.0%	108.9 ± 14.3%	89.4 ± 13.2%
CI	1.2 ± 0.1	1.4 ± 0.2	1.7 ± 1.8	1.1 ± 0.1

### 3.2 Online adaptive plan quality

Two online adaptive sessions (with two separate radiation oncologists) were simulated within the Ethos emulator for all RT intents for all patients. The results of these sessions demonstrated superior adapted plans with superior PTV coverage compared to scheduled plans. Most PTVs and GTVs in Ethos scheduled plans did not achieve clinical goals, whereas a high percentage of PTVs and GTVs achieve clinical goals in the Ethos adapted plans (Table 3). Traditional plan quality metrics for assessing plan conformality, such as R_50%_ and D_2cm_, were superior in Ethos adapted plans with 5.9 ± 2.0 vs 6.6 ± 2.1, and 89.4 ± 13.2% versus 108.9 ± 14.3% in Ethos adapted versus scheduled plans, respectively. CI was closer to the ideal value of 1.0 (1.1 ± 0.1) when compared to the scheduled plans (1.7 ± 1.8). Therefore, Ethos adapted plans were able to better spare normal tissue. Adapted plans would have been selected for treatment rather than scheduled plans in 100% of the on‐couch adaptive sessions.

**FIGURE 4:**
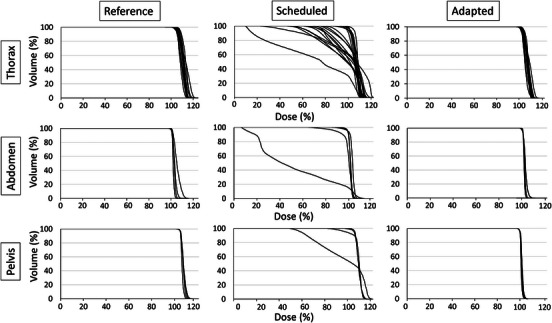
Dose volume histogram (DVH) for 3 RT intents in Ethos (thorax, abdomen, and pelvis) of the patient shown in Figure 3 during the on‐couch adaptation. Adapted plans achieve target coverage goals and generally have improved homogeneity compared to reference plans, whereas scheduled plans are not clinically acceptable due to changes in target shape/location.

**FIGURE 5:**
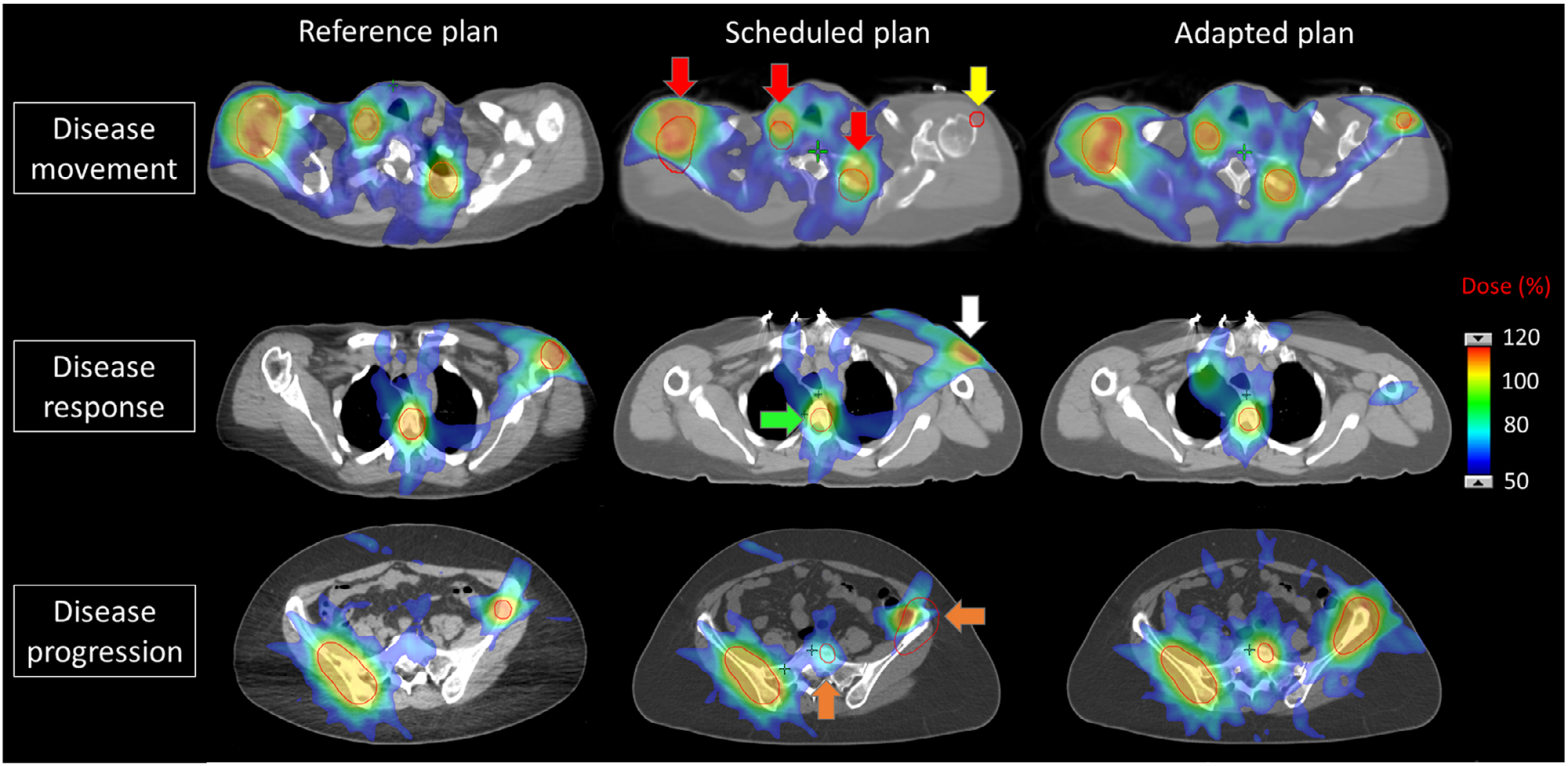
Dose colorwash of the reference, scheduled and adapted Ethos plans for the patient shown in Figure 4 demonstrating three different scenarios: disease movement (in/out of plane), disease response (shrinking target), and disease progression (growing target). Red arrows highlight regions of excessive normal tissue dose due to disease movement, yellow arrow highlights region of target under‐coverage due to movement of the disease to an inferior slice (not pictured), green arrow represents the case of disease regression, white arrow represents the case of disease response (GTV completely eliminated from treatment during the adaption), and orange arrows show the regions of disease progression. Adapted plan accounts for all of these scenarios and successfully optimized the dose to the new anatomy whereas the scheduled plan is not robust to such changes.

Figure 4 shows the DVH for the three RT intents (thorax, abdomen, and pelvis) of the patient with 31 PTVs (target distribution and beam geometries shown in Figure 3) during the on‐couch adaptation. The DVHs of the reference plans showed that all goals were met in initial planning. The Scheduled plans could not meet the goals and for some cases (thorax) were not made available for review or treatment selection during the on‐couch adaptation due to hard‐coded Ethos plan quality checks/requirements for target coverage (e.g., target mean dose <50% Rx). In this particular case, multiple targets failed to meet clinical goals within the acceptable variation for the scheduled plan while the adapted plan succeeded in meeting clinical goals. The percentage of PTVs achieving the goals (acceptable variation) of V_1Gy _> 95% (90%), D_min,0.03cc _> 0.95 Gy (0.90 Gy), and D_0.03cc _< 1.20 Gy (1.25 Gy) were 32.3% (41.9%), 22.6% (32.3%), and 90.3% (100.0%) for the scheduled plan, respectively (versus 100% for all goals in the adapted plan). This plan was available for dosimetric analysis only after exporting the session to eclipse for the purpose of generating a composite from a sum of all RT intents. By comparison, the adapted plans successfully adjusted the dose distributions to cover the new targets as demonstrated by the associated sharp DVH curves indicative of both impressive coverage and homogeneity. Figure 5 shows the dose colorwash of the reference, scheduled and adapted Ethos plans for the same representative patient with 31 targets presenting three scenarios of disease movement from patient setup and/or anatomical changes over time, disease regression and disease progression. Dose colorwash in the reference Ethos plan was conformal to targets. However, target coverage in the scheduled Ethos plan was poor with overtreatment of the normal tissue due to different scenarios such as PTV movement, regression, and progression. Overall, the adapted plans succeeded in optimizing the dose to the new anatomy.

**FIGURE 6:**
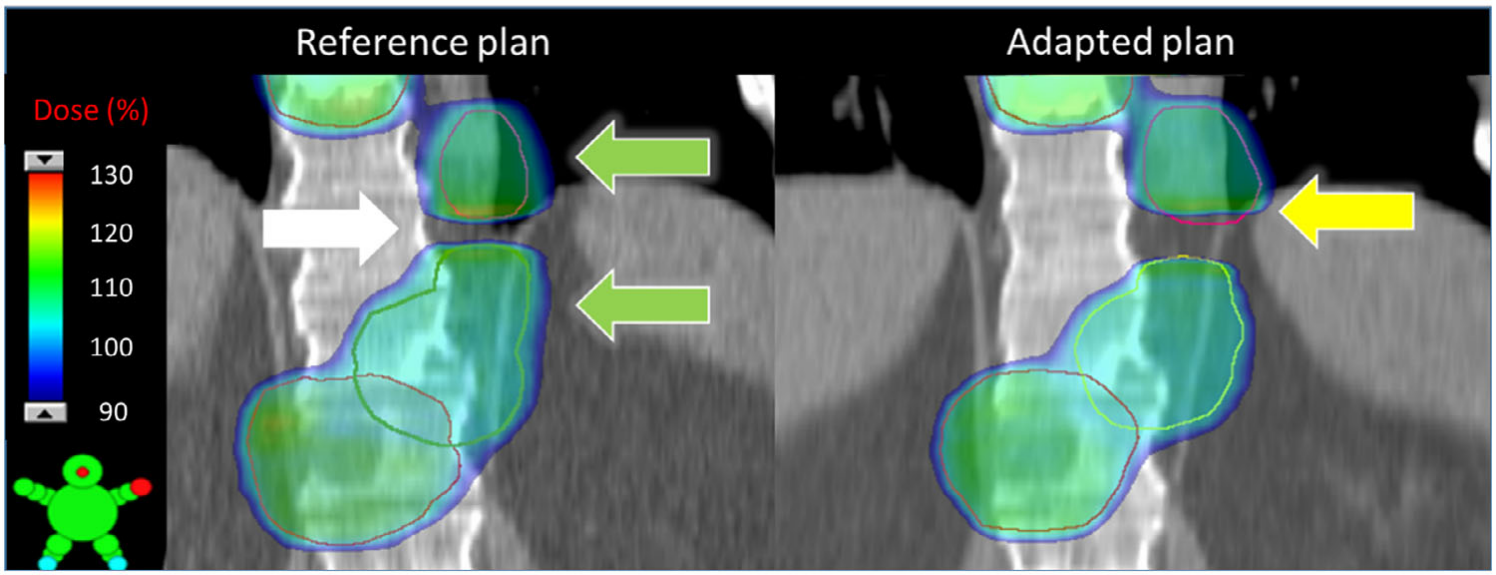
Dose colorwash of the reference (initial) and adapted Ethos plans for a patient showing the junction problem due to 2 adjacent targets (green arrows) in two separate RT intents. White arrow shows the success of the developed initial planning approach in covering the adjacent targets with the prescription dose without hotspot at the junction. Whereas yellow arrow shows how the adapted plan failed to optimize the dose to cover the changes in the anatomy.

Figure 6 shows a dose colorwash of the reference and adapted Ethos plans for the patient exhibiting the dosimetric issue at the junction between two adjacent targets in two independent RT intents. The initial plan (reference plan) succeeded in covering the two adjacent targets with the prescription dose without having hotspots at the junction when the composite dose was analyzed. However, the adapted plan could not adapt the dose to the new targets and resulted in under‐coverage of the inferior part of the PTV in the superior RT intent.

Table 4 shows the timing data for the five components of the adaptive workflow from influencer to plan generation for all 10 patients (24 RT intents). The average duration for influencer generation, influencer editing, target generation, target editing and plan generation (min:sec (minimum‐maximum)) was 01:14 (00:26‐01:52), 02:59 (00:21‐07:53), 01:54 (00:51‐03:55), 13:21 (00:57‐35:55), and 07:08 (03:51‐11:48), respectively. For influencer editing time, one data point that was more than three standard deviations separated from the average was excluded and considered as an outlier. The total duration of the online adaptive workflow, following image acquisition through plan generation, was on average 26:15 (06:43‐57:30). Influencer and target generation were the fastest steps. The majority of influencers and targets required editing for all RT intents. Targets needed more time to edit compared to influencers.

**TABLE 4:** Timing data for all 10 patients (all RT intents) of simulated oART steps.

Duration [min:sec]	Mean	Median	Min	Max
Generate influencers	01:14	01:14	00:26	01:52
Edit influencers	02:59	01:59	00:21	7:53
Generate targets	01:54	01:44	00:51	03:55
Edit targets	13:21	12:36	00:57	35:29
Generate plans	07:08	06:48	03:51	11:48
Total duration	25:39	25:00	06:43	57:30

**FIGURE 7:**
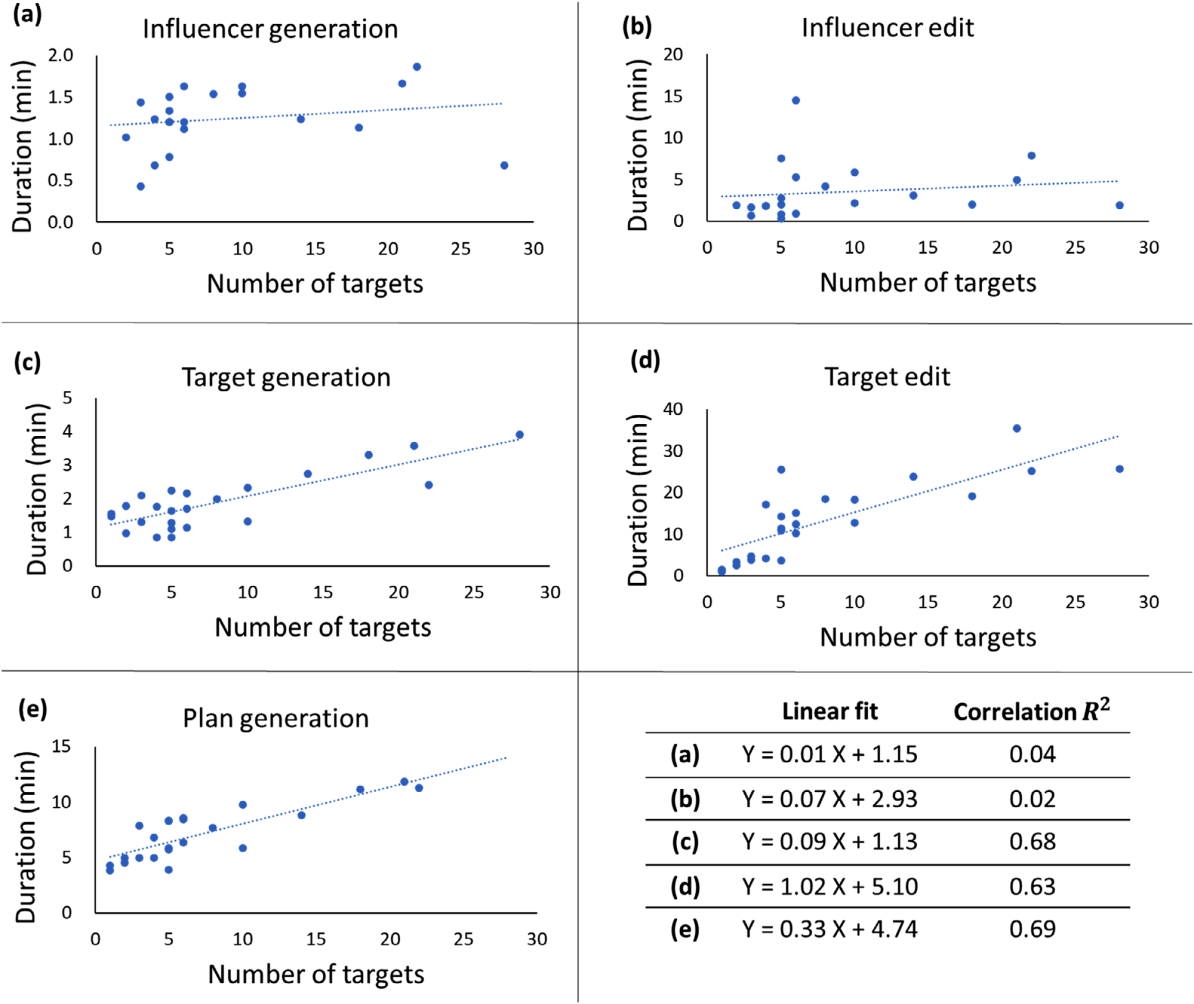
Duration of all steps during the oART as a function of number of targets in a given RT intent with the associated linear fit and correlation. X and Y represent the number of targets and the duration (min), respectively.

Figure 7 presents the duration of all online adaptive workflow components in Ethos as a function of the number of targets including the associated linear fit and correlation. The duration for influencer generation/edit had a slight slope and did not depend on the number of targets with a low correlation coefficient (*R* ^2 ^= 0.04/0.02). In fact, it did depend on the anatomical region/site and so on the influencers (Table 2). The average influencer generation and edit as a function of anatomical site (thorax/abdomen/pelvis) was as follows: 01:27/01:25/00:56 and 05:45/01:23/01:23. In contrast, the duration for target generation/edit and plan generation increased with the number of targets with high correlation coefficients (*R* ^2 ^= 0.68/0.63 and 0.69, respectively). The linear fits provide a model to approximate the time expected for each step in the adaptive workflow (as well as the total time) for a patient based on the number of targets to be treated and thus could facilitate prediction of necessary treatment time‐slot for a given patient.

## 4 DISCUSSION

The use of LDRT to target all sites of metastatic disease might improve immunotherapy response by direct manipulation of the tumor microenvironment at all radiographically evident sites of disease. LDRT has been shown in several preclinical and human trials to have a significant impact on immune cell recruitment^19^ and even radiographic response at the irradiated site.^20^ Poor T cell migration to the tumor microenvironment was a major challenge for effective response to immunotherapy.

The newly developed Ethos CBCT‐guided oART provided semi‐automated planning with AI contouring (currently for abdomen and pelvic regions) in addition to being an online adaptive platform. In this study, the feasibility of novel immunostimulatory LDRT of polymetastatic disease (up to 52 targets) for NSCLC cancer with conventional planning and with the Ethos online adaptative platform was successfully demonstrated. This work developed initial planning strategies to treat all sites of gross disease with LDRT, which can be temporally aligned with systemic immunotherapy administration. These strategies used either conventional planning or the new clinical goal planning paradigm within the Ethos system that was robust to plan adaptation. While the dose calculation algorithms were not exactly the same for conventional planning (using Raystation Monte Carlo) and Ethos planning (using the Acuros XB Linear Boltzmann Transport Equation Solver), the comparison between the two results were assumed to be fair due to the similarity in these algorithms, which has been demonstrated in the literature showing strong agreement with nearly identical dose distributions.^21^ For conventional planning, a general‐purpose clinically commissioned TPS was used. Whereas, for Ethos planning, an emulator software was used for initial planning as well as oART simulations in silico.

For each anatomical site in Ethos, a planning directive template was created and saved with all relevant information for planning including dose prescription, fractionation, normalization, adaptation, structures with their derivations, goals and priorities. For multiple anatomical sites with many structures and goals, use of such templates accelerated the overall planning time and would be useful for true clinical implementation. For mobile targets, the generic 1 cm margin was taken not only to account for set‐up uncertainties but organ motion as well. However, for scenarios where targets are expected to move with breathing motion and have potential to exceed this margin, 4DCT with internal target volume (ITV) approaches or even breath hold could be considered.

Since the patients used in this study were not candidates for RT and thus did not have available simulation CTs, initial planning was done using the CT of the diagnostic PET/CT (instead of a simulation‐CT as commonly used in radiation therapy). The Ethos system (v1.1) was limited to two isocenters in a single RT intent for a maximum combined target length of 38.5 cm, unlike the conventional planning where all targets were optimized in a single plan with multiple isocenters. The plans achieving the highest dosimetric quality were IMRT and VMAT in Ethos and Raystation (e.g., 42 IMRT fields vs. 3 full arc VMAT in Figure 3), respectively. This resulted in multiple RT intents with higher numbers of fields in Ethos compared to the efficiency of a single plan with a maximum of 6 fields (full VMAT arcs) in Raystation. This planning inefficiency was minimized in the workflow developed herein by implementing RT intent templates to streamline initial planning. Additionally, the relatively rapid gantry rotation of Ethos (up to 4 RPM) helps to compensate for the higher number of fixed gantry‐angle IMRT fields. Since neither co‐optimization between different RT intents nor composite plan generation are available in Ethos, there is a significant increase in the planning time for patients with adjacent targets in separate RT intents that may have dosimetric crosstalk between individual plans. This is due to the fact that separate plans for each RT intent must be exported to a separate system (e.g., Eclipse) to allow evaluation of the composite dose distribution (Figures 2 and 6). If this composite dose is unacceptable, new independent plans must be generated in the hopes that the new composite will be acceptable. This necessary trial and error approach is inefficient and time‐consuming. The approach detailed for the single patient in this study with such a junction issue produced an acceptable initial plan but failed to adapt the plan appropriately to variable separation between adjacent targets. Future work must expand upon this to develop an approach that is successful for initial planning and is robust to online adaptive planning. If future versions of Ethos beyond v1.1 incorporate co‐optimization of more than two isocenters for a given RT intent, this may no longer be an issue.

The Ethos emulator provided capabilities to simulate the online adaptive workflow in silico without requiring a fully functioning clinical system. Some steps that would be part of the clinical workflow may be slightly different or not included in the emulator workflow. For example, considerations for patient setup at the machine could not be included in this emulator work, CBCT image acquisition was replaced with software simply loading previously acquired DICOM data, online plan QA and associated timing was not evaluated, and actual beam delivery did not occur. Image acquisition, plan QA and treatment delivery were quantified in a head and neck study and were 1.6 min, 2−3 min, and 1−2 min, respectively.^22^ However, future work will seek to quantify the timing of these components within the unique RT context presented in this work. Additionally, the computer hardware for the emulator software was not identical to that of the clinical system and therefore could affect accuracy of some of the timing data reported in this work when compared to what would be seen in the true clinical Ethos system (assumed to be an overestimation of timing for these steps in this work with improved calculation capabilities in the real clinical system).

In the oART workflow, a subsequent diagnostic scan was used to represent the CBCT of the day or low‐resolution deformation of the initial scan was used to generate an image representing subtle anatomical changes on the day of adaptation (in order to avoid non‐physical warping) and done with a CT of another patient with similar body posture and size in Mirada. Whichever option was used (subsequent diagnostic CT or deformed CT), the image was cropped in the superior/inferior direction to match the field of view (FOV) from a CBCT and to place the image center appropriately (as the CBCT acquisition center is where the isocenter for the online adapted plan is placed within Ethos). In general, this is only an approximation of what would truly be seen for CBCT‐based online adaptive radiotherapy for such patients. However, this approach was found to provide interesting scenarios where disease position changed slightly, patients lost/gained weight, and disease size changed (simulating response or progression). Using these higher image quality substitutes for CBCTs might affect specific portions of the adaptive workflow. Using a lower quality CBCT could affect target delineation and the DIR from the initial scan to the scan of the day that was done behind the scenes which the user cannot control. In results, this might impact the generation of the synthetic CT that in return will affect the structure generation and the dose calculation. Thus, this will affect the accuracy of the results shown in this work compared to the true clinical workflow using the CBCT. However, with the newly developed Hypersight (Varian Medical Systems, Inc., Palo Alto, CA) imaging features, CBCT image quality approaches that of diagnostic CT, making use of CT portion of PET/CT as a pseudo‐CBCT more reasonable.^23^ Nevertheless, future work will seek to address the limitations of the presented work by using prospectively curated data (e.g., CBCTs) more closely representing what would be seen clinically for the proposed methodology.

Dose calculation during online adaptation was based on the synthetic‐CT, which was a DIR of the initial scan to the scan of the day. This DIR was done automatically behind the scenes and the user has no real knowledge or control of this registration, which could affect the structure propagation and the adapted plan dose calculation accuracy and efficiency. In future versions of Ethos, the option to adjust the DIR and/or resultant density map would be useful to improve target propagation which will save some time during the structure review/edit. Alternatively, eventual direct dose calculation on the high‐quality Hypersight CBCTs for Ethos online adaptive planning could address this and will enhance the utility of the treatments developed in this work by more directly accounting for patient changes over a potentially prolonged treatment course. This highlights an additional important proof‐of‐concept indirectly presented in this work. While it is a limitation that we did not have real on‐treatment CBCTs to use for simulated online adaptation in the Ethos emulator, planning on diagnostic CT scans (e.g., from a PET/CT) with eventual online plan adaptation to any changes in patient orientation, anatomy, and disease demonstrates powerful potential for the utility of Ethos in this and other contexts. This makes efficient use of resources and obviates the need for an extra patient visit for a simulation‐CT scan as well as the associated costs and resources for a department associated with this step in the traditional radiotherapy treatment workflow. Similar approaches have been investigated in applications of Ethos for palliative treatments^24^, ^25^, ^26^ and will be made more effective with direct dose calculation on Hypersight CBCTs.

The choice herein to use RT intents with the highest number of influencers which prolong their edit and affect the DIR and target propagation could be further investigated. This could be done by considering RT intents without influencer structures with DIR based on bone anatomy/structures, which could be particularly useful for polymetastatic disease that may be largely localized to bony anatomy.

For a single case, during the adaptive workflow, the thorax scheduled plan could not be generated as a treatment option due to significant clinical goal violations (Figure 4). This could be due to global patient anatomical/positioning differences and changes in target structures and positions. This emphasized the importance of the online adaptation where a geographic miss of one or more targets would become increasingly likely over time without adaptation.

Online adapted plans were superior compared to scheduled plans in terms of target coverage and OAR sparing for all plans (Figure 5) except for one patient with adjacent targets in independent RT intents (Figure 6). Although, the initial planning approach succeeded in achieving the desired coverage without hotspot at the junction level, the independent optimization of two RT intents without knowledge of the contribution of dose from one intent to the other failed in generating an acceptable adapted plan. Ideally, future versions of Ethos will enable incorporation of greater than 2 isocenters in a single RT intent. Without this feature, future work will continue to investigate optimization approaches to handle dose gradients at the junction of adjacent targets that is robust to potential anatomic changes and subsequent plan adaptation.

Initial plan normalization was used for 50% of the RT intents to achieve the combination of all PTVs D_min,0.03cc _= 95%. In the current version of Ethos (v1.1), this normalization factor must be set prior to initial plan generation and will be applied to the online adapted plans which may or may not be needed (and in some scenarios may have unwanted dosimetric effects on the adapted plan, such as excessive hot spots to achieve coverage). Therefore, the option of setting the normalization as a final step after initial and online adapted plan generation would be useful in the future versions of Ethos.

## 5 CONCLUSION

This study showed the general technical feasibility of novel immunostimulatory LDRT (1 Gy) to comprehensively treat all sites of polymetastatic disease in stage IV NSCLC cancer patients, which could provide treatment options for patients that traditionally would not be considered candidates for RT. Initial planning was shown to be feasible with both conventional planning/treatment (Raystation/Halcyon) and a novel AI‐driven online adaptive RT platform (Ethos), and all plans in both contexts were able to meet all clinical goals (within the acceptable variation). Beam geometry of Ethos plans consisted of a higher number of fixed‐gantry IMRT fields compared to Raystation plans where 3 to 6 full VMAT arcs were used. However, the fast treatment delivery with Ethos makes delivery of a high number of IMRT beams logistically reasonable. While both Ethos and Raystation plans were able to achieve clinical goals within the acceptable variation, Ethos plans were dosimetrically superior, sparing more normal tissue with homogeneous target coverage. This is illustrated by the higher percentage of PTVs achieving goals combined with lower R_50%_ and D_2cm_ values as well as CI values closer to 1 for Ethos plans versus Raystation plans. The semi‐automated goal‐based planning features in Ethos also made initial planning logistically less complex, requiring less user input and time. A current limitation of Ethos is the inability to co‐optimize more than 2 isocenters in a single plan. This issue is particularly relevant when irradiating disease distributed throughout a patient's entire body and was illustrated with a representative patient. Although the composite plan for the patient with two adjacent targets in separate RT intents successfully covered the targets conformally with the prescription dose in the initial composite plan, this failed to be the case in the composite adapted plan. Therefore, more investigation is needed to determine an approach to handle such scenarios that will be robust to changes over the course of treatment. For the online adaptation, Ethos scheduled (non‐adapted) plans failed to achieve clinical goals due to anatomical changes and disease size/shape/location changes, whereas Ethos adapted plans conformally covered all PTVs with the prescription dose homogeneously while sparing normal tissue within a newly developed efficient online adaptive workflow.

## AUTHOR CONTRIBUTIONS

All of the above listed authors contributed directly to the intellectual content of the paper including work design and acquisition of data, writing/editing the manuscript, and final approval of this version.

## CONFLICT OF INTEREST STATEMENT

The authors declare no conflicts of interest.
